# Single nuclear RNA sequencing shows altered microglial and astrocytic functions in post-mortem Parkinson’s disease tissue

**DOI:** 10.1186/s12863-026-01462-2

**Published:** 2026-07-04

**Authors:** Indra Roy, Michael Fiorini, Rhalena A. Thomas, Georgina Jimenez Ambriz, Sali Farhan, Valerio E. C. Piscopo, Thomas M. Durcan

**Affiliations:** 1 The Neuro’s Early Drug Discovery Unit (EDDU), McGill University, Montreal, Canada; 2 Neuro-CERVO Alliance for Drug Discovery in Brain Diseases, McGill University, Montreal, Canada; 3 The Neuro Bioinformatics Core, McGill University, Montreal, Canada; 4 Department of Human Genetics, McGill University, Montreal, Canada

**Keywords:** Parkinson’s disease, Single-nuclear RNA sequencing, Microglia, Astrocytes, Glial reactivity, Differential gene expression (DGE), Gene ontology (GO) molecular functions, Functional pathway analysis

## Abstract

**Background:**

Parkinson’s disease (PD) is a neurodegenerative disease marked by a progressive loss of dopaminergic neurons in the substantia nigra pars compacta (SNpc) and formation of misfolded protein aggregates. A growing body of research has implicated glial cell dysfunction in PD etiology, including the concentration of activated glial cells around protein aggregates in post-mortem tissue. A disruption in the balance of pro- and anti-inflammatory immune response functions of microglia and astrocytes is believed to contribute towards neuronal degeneration as the disease progresses. However, the molecular mechanisms remain unclear. To shed light on the role of microglia and astrocytes in PD, this study analyzes three public single nuclear RNA sequencing datasets of the SNpc from patient and control post-mortem brains to identify altered molecular pathways in PD.

**Results:**

Both astrocytes and microglia show significant upregulation of heat shock binding and misfolded protein response pathways, likely reflecting a response to accumulating protein aggregates. Additionally, both cell types show decreased expression of genes associated with receptor functions; for microglia this included cytokine receptor genes such as IL21R, IL4R, and IFI44L. Genes associated with resting state microglia and non-inflammatory reactive state microglia were downregulated in PD microglia, including P2RY13, RSAD2, CSF2RA, CSF3R, and CX3CR1. Concurrently, astrocytes and microglia both show decreased expression of genes associated with neurotransmitter receptor functions that include glutamate and other ion channel receptors, suggesting a loss of neuron-glia communication in later disease stages.

**Conclusions:**

Taken together, our findings imply that astrocytes and microglia respond to protein misfolding pathology in PD by upregulating chaperone protein folding functions. Additionally, the profile of upregulated functions implies that both cell types are under increased energetic demand. The downregulation of neurotransmitter and channel receptor functions in both cell types indicates that neuron-glia communication and other supportive functions may be lost in favour of autophagic, protein-clearance mechanisms in PD microglia and astrocytes.

**Supplementary Information:**

The online version contains supplementary material available at https://doi.org/10.1186/s12863-026-01462-2.

## Background

Several neurodegenerative diseases (NDDs), including Alzheimer’s disease (AD) and Parkinson’s disease (PD), are characterized both as proteinopathies, marked by aberrant post-transcriptional modifications and protein aggregates [1, 2], and as neuroinflammatory disorders involving reactive glial cells [3–5]. Although the two mechanisms are hypothesized to overlap [5–8], the exact pathways by which they contribute to neurodegeneration remain unclear. A recent review suggested that glial cells maintain a balance of both pro- and anti-inflammatory functions in the brain and that disrupting this balance promotes the progressive loss of neurons observed across NDDs [9]. Astrocytes, microglia, and infiltrating peripheral T cells collectively promote an inflammatory cascade that can be both pathogenic and protective in these NDDs [10].

Studies exploring the role of glial involvement in NDDs have expanded in recent decades. Evidence of glial activation in early disease stages has been obtained through positron emission tomography (PET) imaging studies, indicating potential causative or exacerbating roles of microglia and astrocytes. Radioligand-labeled translocator protein (TSPO), which marks primarily reactive microglia (and to some extent astrocytes), is increased in disease-relevant areas in both AD and PD [11–13]. Consistent with these findings, post-mortem analyzes reveal increased reactive microglia (Iba-1^+^/HLA-DR^+^/CD11b^+^) around amyloid-β (Aβ) plaques [14], and elevated densities of reactive microglia (HLA-DR^+^) in the substantia nigra *pars compacta* (SNpc) of PD patients, particularly surrounding synuclein deposits [15].

Reactive astrocytes are also observed in disease relevant areas from human post-mortem tissue in several NDDs, including the prefrontal cortex in AD [16], motor cortex in amyotrophic lateral sclerosis (ALS) [17], and SNpc in PD [18]. Functional studies further highlight astrocyte dysregulation, as astrocytes cultured from *LRRK2* mutant familial PD patient-derived induced pluripotent stem cells (iPSCs) showed dysfunctional autophagy, increased reactivity, and impaired ability to phagocytose α-synuclein (α-syn) aggregates [19].

Microglial response to α-syn appears to be context-dependent. Neuronal α-syn can activate microglia via toll-like receptor 4 (TLR4), accompanied by upregulation of autophagy receptors through the nuclear factor kappa B (NF-κB) pathway in rat primary microglial cultures [19]. Under physiological conditions, such activation may be protective by promoting clearance of misfolded α-syn. However, α-syn also induces pro-inflammatory cytokine production in iPSC-derived microglia [20], with dose-dependent increases in IL-1β and TNF observed in both human and mouse microglial models [20, 21]. These findings suggest that persistent accumulation of misfolded protein aggregates may drive pathology through chronic neuroinflammation.

In addition to inflammatory signaling, glial cells may actively contribute to the propagation of pathogenic proteins. Recent studies using humanized AD animal models indicate that microglial activation is responsible for spreading tau pathology, potentially through the release of partially digested protein fragments via phagosomal pathways [16]. In addition, microglia-derived exosomes have been implicated in the prion-like transmission of α-syn to neurons [22], and a similar glia-to-neuron transmission of α-syn was observed in astrocyte-neuron co-culture models [23]. Together, these findings support a role for glial cells in facilitating the spread of α-syn, thereby contributing to neuronal cytotoxicity in PD and other NDDs [23, 24].

Despite growing evidence, the molecular mechanisms underlying PD remain incompletely characterized, and the role of glial cells in neurodegeneration is still debated. The literature reports contradictory conclusions regarding the role of microglia in NDDs [9, 20–22], with similar inconsistencies observed for reactive astrocytes [23, 24]. These discrepancies likely reflect the substantial phenotypic heterogeneity exhibited by both glial types. Earlier frameworks attempted to categorize astrocytes and microglia as “pro-inflammatory” (reactive) and “anti-inflammatory” states. However, this binary conceptualization has become increasingly inadequate as the context-dependent nature of immune signaling has emerged. For example, IL-10 is often described as anti-inflammatory due to its suppression of pro-inflammatory cytokines such as TNF-α, IL-1β, and IL-6; yet prolonged activation of IL-10 can also trigger a resurgent increase in IL-1β and IL-6, contributing to pro-inflammatory processes [25, 26]. Furthermore, advances in single-cell RNA sequencing have revealed that both astrocytes and microglia can display a variety of activation states that are challenging to categorize within a spectrum of reactivity [27, 28]. This functional diversity complicates efforts to define precise contributions of glial cells to PD pathogenesis.

In this work, we aim to delineate functional alterations across microglia and astrocytes by analyzing three publicly available single nuclear RNA sequencing (snRNAseq) datasets derived from human post-mortem midbrain tissue of idiopathic PD patients and age-matched controls, generated by Smajić et al. (n_PD_ = 5, n_control_ = 6), Kamath et al. (n_PD_ = 6, n_control_ = 8), and Wang et al. (n_PD_ = 23, n_control_ = 9) [29–31]. We perform differential gene expression (DGE) analysis and subsequent pathway analysis to identify genes associated with certain molecular functional pathways that are altered in PD microglia and astrocytes. We hypothesize that altered gene expression in patient-derived glial cells will reveal dysregulated molecular functions that contribute to proteinopathy.

## Methods

1. Data acquisition and processing: For the Kamath et al. [29] dataset, we obtained the filtered gene expression matrix from the Broad Institute’s Single Cell Portal, and extracted cells annotated as astrocytes or microglia in separate data objects. For quality control purposes, Kamath et al. removed cells with >10% mitochondrial RNA and those with significantly lower or higher UMI counts relative to the rest of the dataset. The cell-type annotations provided by Kamath et al. were obtained from the UMAP.tsv files available on their portal, and cell barcodes were matched to incorporate these annotations as metadata into our Seurat object [32]. The original dataset includes samples from patients with Lewy body dementia, which were excluded from our analysis.

For the Wang et al. [31] and Smajić et al. [30] datasets, the raw data was downloaded from Gene Expression Omnibus (GEO; GSE157783 and GSE184950, respectively). The raw sequencing data was processed using the scRNAbox pipeline [33]. FASTQ files were aligned to the human genome (GRCh38), and the expression count matrix, feature and single-cell barcode files were generated using CellRanger v.7. For Smajić et al., quality control was performed to retain cells expressing a minimum of 1,000 unique gene transcripts and <25% mitochondrial RNA. For Wang et al., cells expressing 200–2,500 unique gene transcripts and <10% mitochondrial RNA were retained. PD patients with frontotemporal dementia from the Wang et al. dataset were removed from the analysis. For both datasets, doublets were identified using DoubletFinder [33] and removed using step 4 of the scRNAbox pipeline. Dataset-specific samples were integrated using the Seurat V4 integration functions [33]. Cells from the integrated data object were subsequently clustered: the top 3,000 and 2,500 most variable features for Smajić et al. and Wang et al., respectively, were input into principal component analysis. The top 30 and 20 principal components for Smajić et al. and Wang et al., respectively, were used as input for generating a shared nearest neighbour graph (SNN) and uniform manifold approximation and projection (UMAP) visualization. Clusters were identified using the Leiden network detection algorithm with the SNN graph as the input and run for a range of clustering resolutions; clustering resolutions of 0.2 and 0.8 for Smajić et al. and Wang et al., respectively, were selected for cell type annotation. Annotations for the Smajić et al. and Wang et al. datasets were generated using step 7.1 of the scRNAbox pipeline, in which Seurat’s *FindAllMarkers* function was used to identify cluster-specific differentially expressed genes (DEGs), followed by enrichment testing against three curated libraries describing cell-type marker genes [34–36] using the EnrichR tool [37]. Additionally, reference-based annotation was performed to project cell-type annotations from the Kamath et al. reference dataset onto the Wang et al. query dataset, using step 7.3 of the scRNAbox pipeline, which incorporates Seurat’s *FindTransferAnchors* and *TransferData* functions.

2. Cell population testing: A proportionality test was performed to identify any contributions of disease to cell type numbers. ScProportion [38] was used to perform permutation tests comparing cell-type proportions between the PD and control groups, by evaluating observed differences against a null distribution generated through random shuffling of group labels for each cell type. To validate the findings of the proportionality test, a second, hierarchical Bayesian proportionality test was also implemented using the brms package [39]. The posterior mean log2 fold-change in the relative abundance/composition of each cell type is estimated in PD versus control using a Bayesian multinomial model of cell-type counts in each group.
3. Differential gene expression (DGE) analysis: DGE analysis was performed using Seurat’s *FindMarkers* function, contrasting PD and control groups with a MAST mixed-effects model [40]. Sex and donor ID were included as latent variables, and statistical significance was defined using an adjusted *p*-value threshold of 0.05 following Benjamini-Hochberg correction. Separate MAST mixed-effects models were computed for each dataset and cell type. A split control permutation test was performed on the Kamath et al. dataset as a negative control of our MAST method (Supplementary figures 1c and d). As a complementary method, we performed pseudo-bulk analysis across all three datasets together, in which donor-level aggregate expression was calculated separately for each dataset to prevent bias due variations across datasets (e.g., due to variations in read depth, cells per donor, etc.). Aggregated count matrices were combined and analyzed using edgeR [41]. Lowly-expressed genes were filtered using filterByExpr, followed by TMM normalization to account for library size differences. DGE was calculated using a generalized linear model with disease status and sex as covariates, and significance was calculated using quasi-likelihood F-tests. A split control permutation test of this method was also performed using control donors from Kamath et al. and Wang et al. datasets (Supplementary figures 1a and b). Full DGE lists are made available in supplementary tables 1-6.
4. Functional enrichment analysis: Gene set enrichment analysis (GSEA) was performed to functionally characterize altered gene expression between PD and control. All genes were ordered based on the log fold change (logFC) values output from the MAST mixed-effects and the pseudo-bulk models, independently. The resulting ranked lists were input into ClusterProfiler’s [42] *gseGO* function to compute enrichment across the Gene Ontology (GO) Molecular Function library, identifying functions that are significantly enriched among up- or down-regulated genes. This approach determines whether genes associated with specific molecular functions show coordinated up- or down-regulation in PD. In our results, we emphasize the DGE and molecular functions that are preserved in the cross-dataset analysis. Full GSEA results are made available in Supplementary tables 7-12.

## Results

### Microglia and astrocyte abundances are not altered in PD

**Fig. 1:**
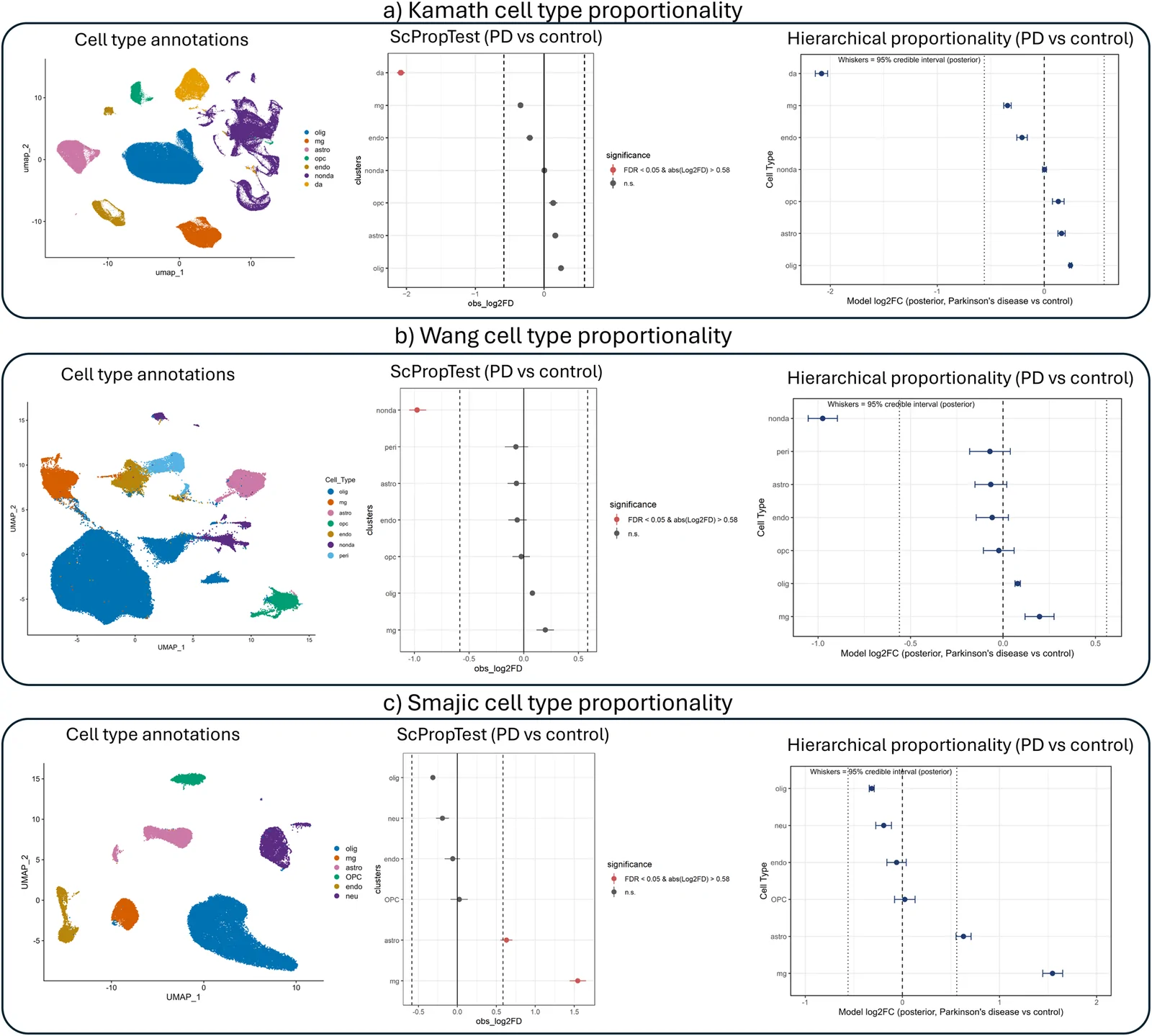
UMAP distribution of all cell types contained in the (**a**) Kamath et al., (**b**) Wang et al., and (**c**) Smajić et al. datasets on the left. Cell type abundance calculated using two separate methods are shown for each dataset. The change in observed cell type abundance in PD samples is calculated against predicted abundance based on cell proportions in control populations using a null distribution model (ScProptest) or a Bayesian multinomial model (brms). Whiskers around the observed log2FC show the 95% confidence interval of the change. Abbreviations: dopaminergic neurons (da), non-dopaminergic neurons (nonda), neurons (neu), oligodendrocytes (olig), oligodendrocyte progenitor cells (OPC), microglia (mg), astrocytes (astro)

One way to determine whether cell types are impacted in disease is to perform a proportionality test, which evaluates whether specific cell types are over- or under-represented in diseased tissue relative to their proportions in controls. A lower-than-predicted cell number may suggest a cell type is vulnerable and thus selectively lost in disease. A higher-than-expected number suggests that these cells are upregulated in disease states, either as part of the pathology or as a compensatory response. As expected, in the Kamath et al. dataset, the proportion of DA neurons was significantly lower in PD than in controls (obs. log2FC ~ −2.3, *p* < 0.01; Fig. 1a). Due to the relative sparsity of DA neurons in the SNpc and potential loss due to sample preparation, DA neurons are difficult to capture in sequencing unless they are specifically enriched for. While Kamath et al. did this, Wang et al. and Smajić et al. did not, so we were unable to annotate DA neurons in these datasets. Nonetheless, the proportion of non-DA neurons was lower in PD in the Wang et al. dataset (obs. log2FC ~ −0.95, *p* < 0.01; Fig. 1b). The proportions of microglia and astrocytes did not differ (obs. log2FC between −0.56 and 0.56) between PD and controls in the Kamath et al. and Wang et al. datasets (Figs. 1a and 1b); however, in the Smajić et al. dataset, the proportion of microglia was significantly higher in PD (obs. log2FC ~ 1.6, *p* < 0.01; Fig. 1c). Given the smaller size of the Smajić et al. dataset, we attribute more accuracy in this regard to the other datasets. We conclude that microglia and astrocytes in PD neither show vulnerability to degeneration nor proliferate in greater numbers.

### Microglia and astrocytes upregulate chaperone protein binding functions in PD

Next, we computed DGE between PD and controls in microglia and astrocytes. The resulting fold-change values between PD and control were then used to order genes for input to GSEA to identify molecular functions with altered activity in PD cells. For the Smajić et al. dataset, no genes met significance when analyzed independently; thus, this dataset was only included in the donor-level pseudo-bulk model that incorporates all three datasets. We report results that appear in all three DGE contrasts: 1) mixed-effects MAST model applied to Kamath et al., 2) mixed-effects MAST model applied to Wang et al., and 3) pseudo-bulk-based DGE analysis from the aggregated expression matrices from all three datasets.

**Fig. 2:**
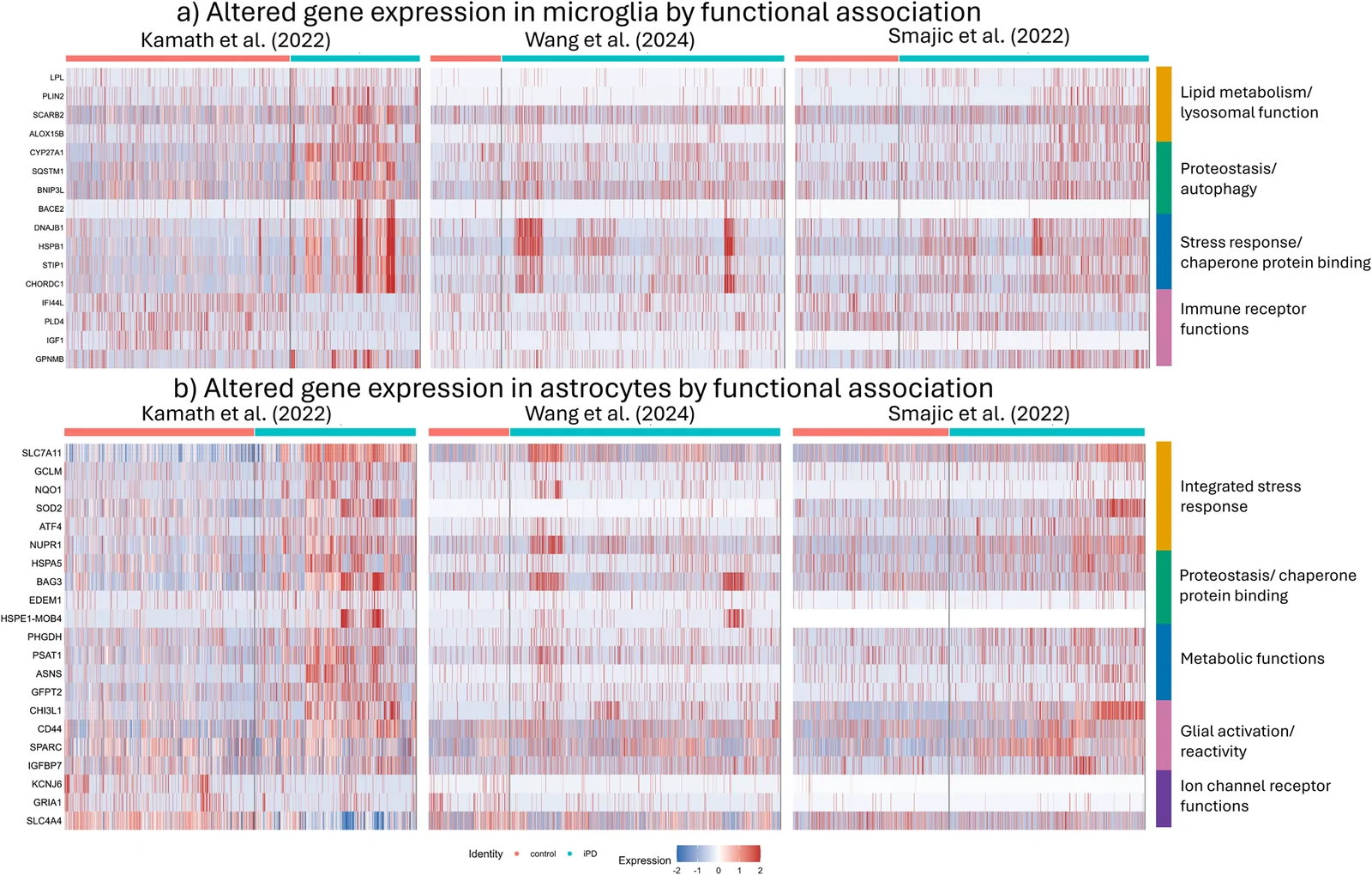
Heatmaps show the differential expression of selected genes of interest across all three datasets in (**a**) microglia and (**b**) astrocytes. Genes are groups by functional association relevant to the respective cell type

**Fig. 3:**
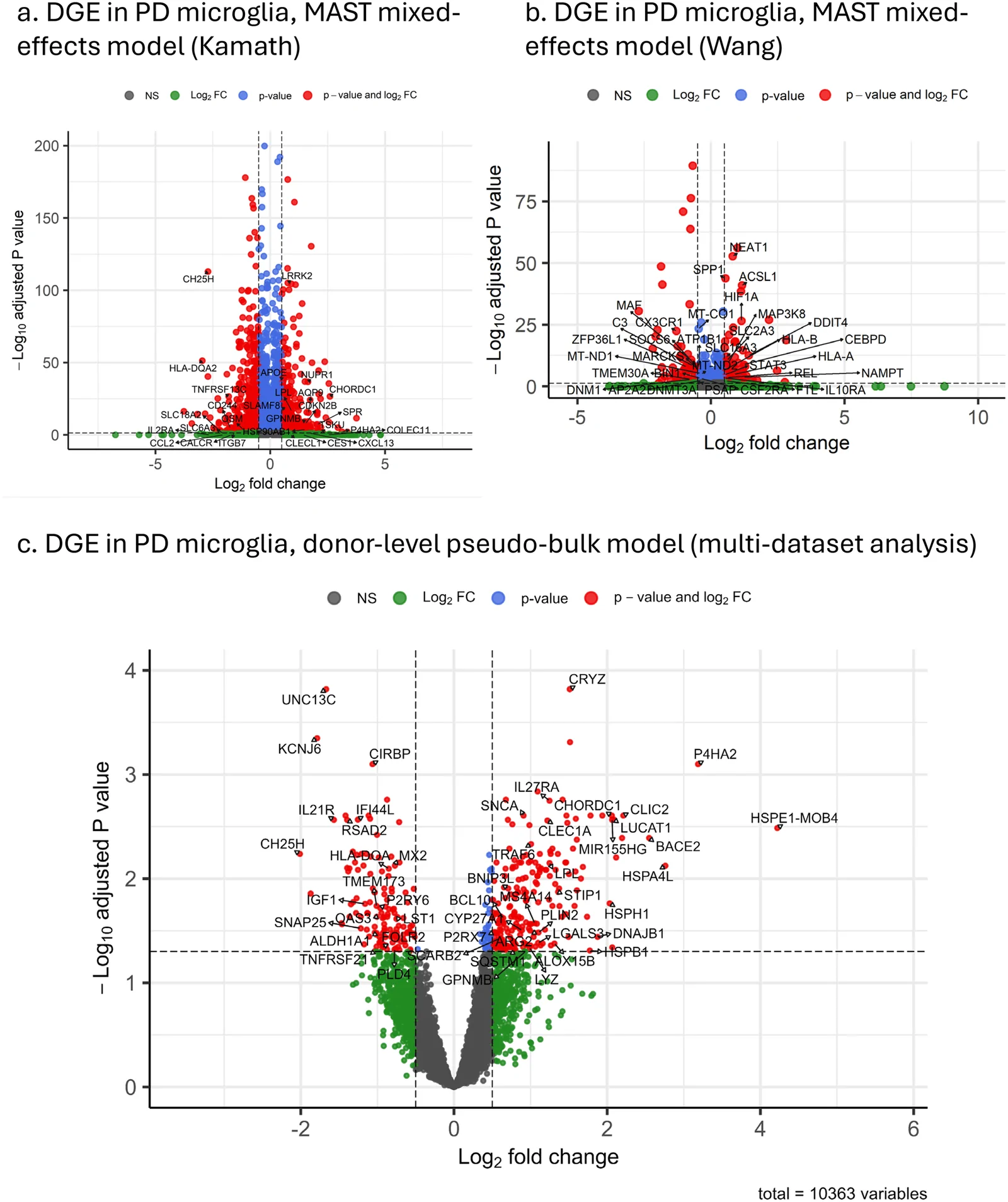
Volcano plots of DGE in PD microglia analyzed using the MAST mixed-effects model within the (**a**) Kamath et al. And (**b**) Wang et al. datasets. (**c**) DGE in PD microglia analyzed using the donor-level pseudo-bulk model across all three datasets combined

**Fig. 4:**
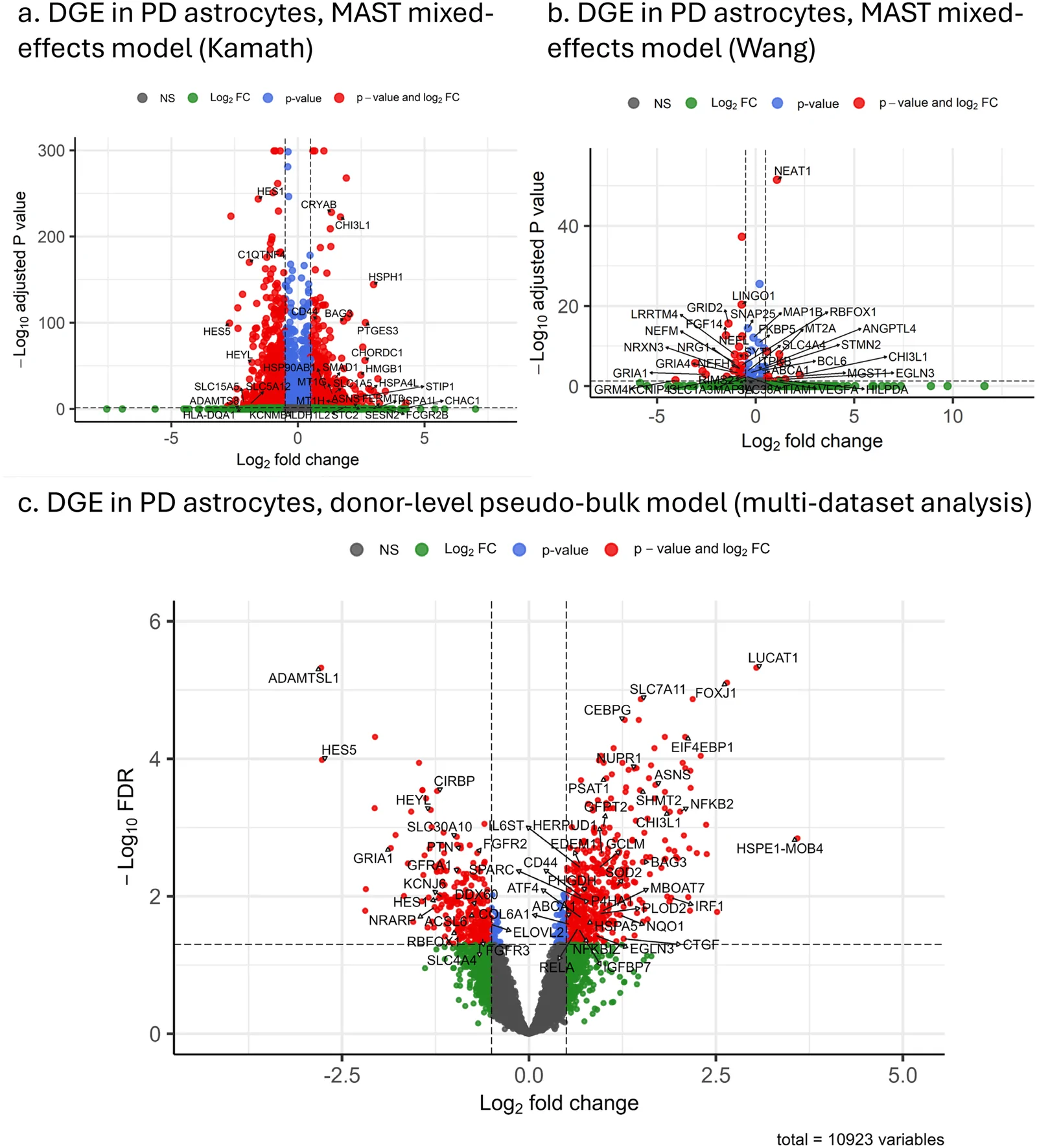
Volcano plots of DGE in PD astrocytes analyzed using the MAST mixed-effects model within the (**a**) Kamath et al. And (**b**) Wang et al. datasets. (**c**) DGE in PD astrocytes analyzed using the donor-level pseudo-bulk model across all three datasets combined

**Fig. 5:**
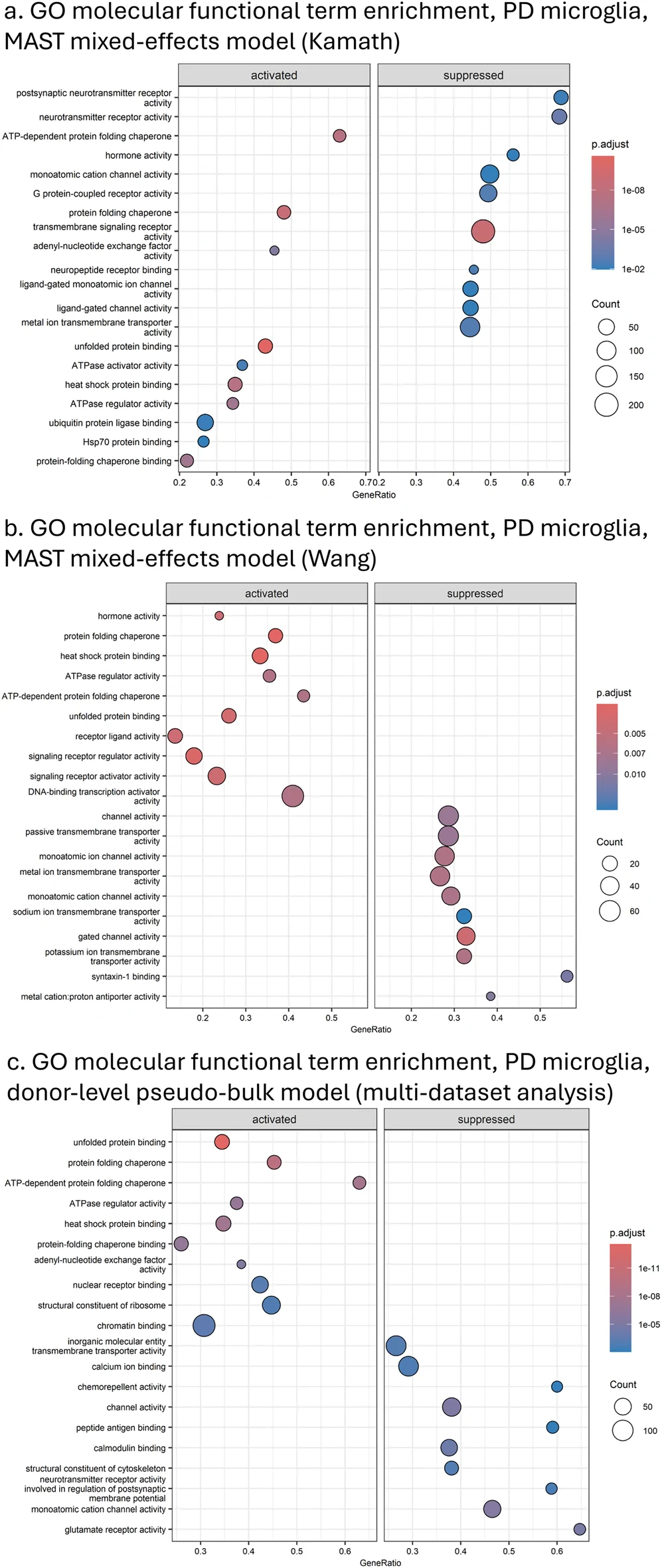
GSEA of the most altered GO molecular functional terms faceted by upregulated and downregulated functions in PD microglia in the (**a**) Kamath et al. and (**b**) Wang et al. datasets, based on the DGE results of the MAST mixed-effects model. (**c**) GSEA of the most altered GO molecular functional terms faceted by upregulated and downregulated functions in PD microglia based on the DGE results of the donor-level pseudo-bulk model across all three datasets combined

**Fig. 6:**
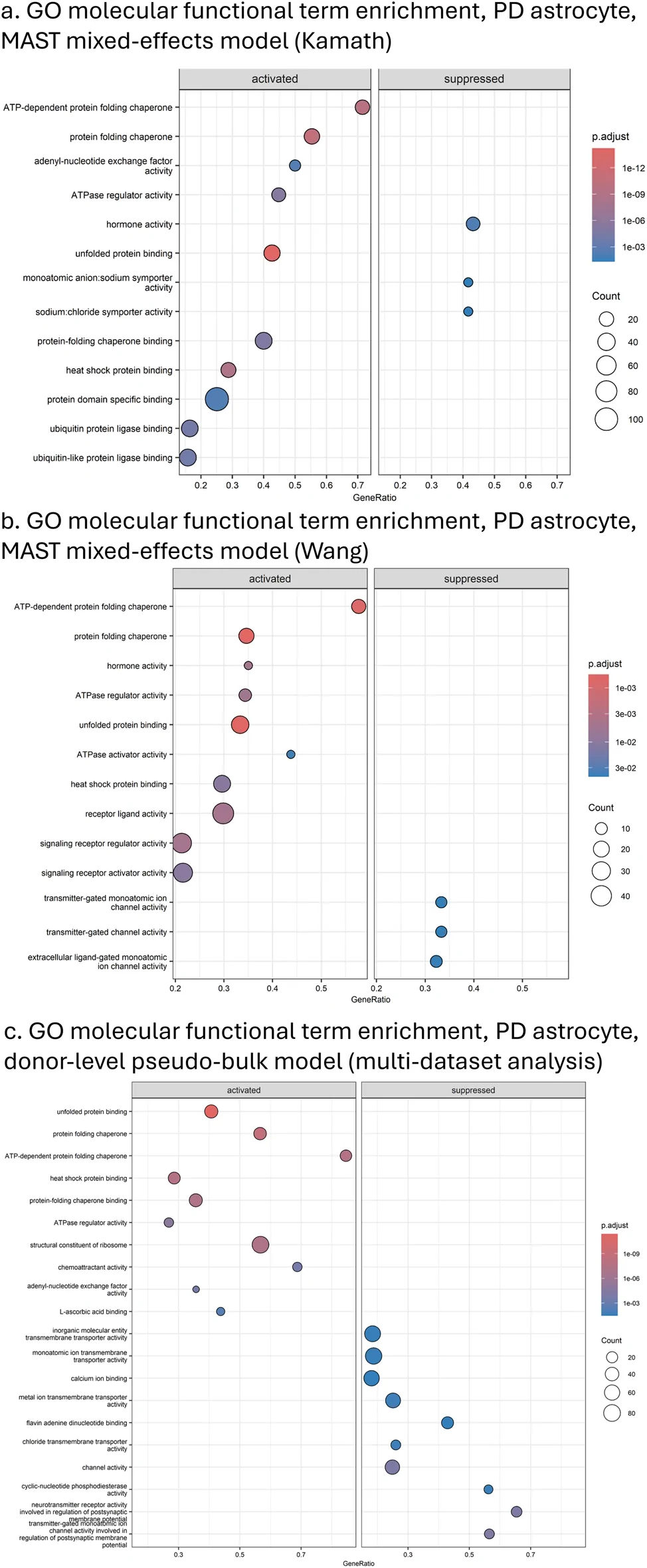
GSEA of the most altered GO molecular functional terms faceted by upregulated and downregulated functions in PD astrocytes in the (**a**) Kamath et al. and (**b**) Wang et al. datasets, based on the DGE results of the MAST mixed-effects model. (**c**) GSEA of the most altered GO molecular functional terms faceted by upregulated and downregulated functions in PD astrocytes based on the DGE results of the donor-level pseudo-bulk model across all three datasets combined

Our analysis suggests that both astrocytes and microglia respond to pathological protein misfolding in PD. Genes encoding heat shock proteins (HSPs) and chaperone proteins dominated the top upregulated genes in both astrocytes and microglia, including *HSPH1*, *DNAJA1*, *STIP1*, *AHSA1*, and *BAG3* (Figs. 2, 3, 4). *BAG3* is an established PD risk gene in GWAS studies [43, 44] and has been previously shown to have increased expression in PD glial cells [45]. In mouse microglial cells, *BAG3* was shown to promote clearance of α-syn fibrils and autophagy in an inflammatory environment while suppressing NLRP3 inflammasome formation [46, 47]. Furthermore, genes involved in cellular stress response such as *CHORDC1*, *NUFIP2*, and *CACYBP* suggest that microglia and astrocytes are responding to neurodegeneration (Figs. 3 and 4). Consistently, heat shock protein binding (GO:0037312) and protein folding chaperone (GO:0044183) were among the top activated GO terms in both cell types, and this finding remains significant in the MAST mixed-effects analyses of Kamath et al. and Wang et al. datasets, as well as in the combined pseudo-bulk analysis of all three datasets (Figs. 5 and 6). Together, these findings suggest a coordinated upregulation of protein folding pathways across glial cells, consistent with a compensatory response to the accumulation of misfolded and aggregated proteins in PD.

### PD microglia and astrocytes demonstrate increased ATP-dependent energy demand

In both astrocytes and microglia, the consistent upregulation of genes encoding HSPs was paired with enrichment of functional terms associated with ATP-dependent energy demand, including ATP-dependent protein folding chaperone (GO:0140662) and ATPase regulator activity (GO:0060590), likely reflecting an increased energetic demand for protein folding and quality control (Figs. 5 and 6). While an elevated energetic burden in PD glia does not directly demonstrate altered mitochondrial function, it is consistent with an increased reliance on mitochondrial ATP production and may reflect increased mitochondrial load or associated stress. This is supported by several consistently upregulated genes identified in the multi-dataset pseudo-bulk analysis (Figs. 3 and 4), including the *HSPE1–MOB4* readthrough transcript, mitochondrial ribosomal genes (*MRPS6*, *MRPL18*), and *BNIP3L*, all of which are associated with mitochondrial protein folding, translation, and quality control processes [48, 49]. Notably, *HSPE1* encodes a mitochondrial co-chaperonin involved in protein folding [48], and the prominence of the *HSPE1–MOB4* readthrough transcript likely reflects increased activity at this locus rather than a specific function of the readthrough transcript. Together, these findings suggest that microglia and astrocytes are under increased energetic demand as they respond to cellular stress associated with protein aggregation and neurodegeneration in the midbrain.

### PD microglia suppress receptor-mediated functions, including cytokine receptor functions

Increased expression of HSPs and the associated chaperone folding genes suggests increased cellular stress. Previous studies posited that increased glial activation occurs early in PD, with several PET studies showing an increase in TSPO-radioligand-labeled cellular activity in recently diagnosed PD patients [11, 12]. Studies with cultured microglia from various sources, including rat and mouse primary microglia and human iPSC-derived microglia cells have also shown increased secretion of pro-inflammatory cytokines in response to α-syn fibrils, through NLRP3- and TLR4-mediated activation [50–53]. In this analysis, we found that peptide antigen binding (GO:0042605) was among the top downregulated pathways in PD microglia (Fig. 5). Additionally, neurotransmitter receptor activity (GO:0030594) and glutamate receptor activity (GO:0008066) emerged as strongly downregulated pathways (Fig. 5c) enriched by numerous genes such as *GRIA1*, *GRIK1/2*, *UNC13C,* and *KCNJ6* (Fig. 3c).

The downregulation of peptide antigen binding is enriched by several HLA genes, including *HLA-DOA,* which is an MHC class II receptor regulator and was among the most significantly downregulated genes in PD microglia (Fig. 3). This is accompanied by decreased expression of cytokine receptor genes *IL21R*, *IL4R*, *TNFRSF21*, and several interferon genes such as *IFI44L*, *RSAD2*, and *MX2* (Fig. 2a). Notably, phagocytosis-signalling receptor *P2RY6* is also decreased in PD microglia, suggesting further impairment in microglial function. In addition to the immune receptor genes, several genes that promote microglial surveillance, motility, and response to pattern- or damage-associated markers (PAM/DAMs) are also downregulated, including *CX3CR1*, *CSF2RA*, *CSF3R,* and *P2RY13*. The downregulation of these genes, particularly receptors for colony-stimulating factors suggests impaired ability of microglia to respond to damage and inflammation in PD. Thus, our analysis supports the notion that microglial homeostasis is disrupted in PD, particularly affecting the different microglial states responsible for responding to damage and resolving inflammation and stress-response cascades. One notable exception to this pattern is the increased expression of *GPNMB* in PD microglia (Fig. 2a), which is typically associated with anti-inflammatory response in macrophage-like cells. This could indicate that earlier attempts at damage resolution from responding microglial cells may simply become ineffective or inadequate over time, despite increased transcription at the RNA level.

### PD astrocytes and microglia show neurotransmitter and channel receptor dysfunction

Under physiological conditions, both astrocytes and microglia have supportive roles in neurotransmitter communication [54]. This is particularly true for astrocytes, which regulate neural cell signalling and synaptic transmission through their extensive network of neurotransmitter channels (such as glutamate and GABA) and voltage-gated ion channels (such as Ca2+, Na+, and K+ channels) [55]. In our analysis of donor-level pseudo-bulk DGE across all three datasets, neurotransmitter receptor activity involved in the regulation of post-synaptic membrane potential (GO:0099529) emerges as a key downregulated function in both microglia and astrocytes (Figs. 5c and 6c). In microglia, downregulated receptor activity also includes glutamate receptor activity (GO:0008066), likely representing the loss of microglial ability to modulate excitatory neurotransmission (Fig. 5c). In astrocytes, many of the downregulated functions are associated with ion channel activity or ionic transmembrane transporter functions, emphasizing the loss of a key astrocytic supportive function.

## Discussion

Inflammation is a known pathological feature of numerous neurodegenerative disorders, including PD, and its interaction with proteinopathy has been characterized with limited success and mixed results in cell cultures and animal models. In this study, we leveraged three large public snRNAseq datasets from human post-mortem midbrain tissue to investigate altered molecular signatures in astrocytes and microglia, two key glial cell types implicated in PD, with the aim of identifying additional degenerative mechanisms.

Our findings show that HSP- and misfolded-protein binding functions were among the most upregulated functions in PD glia. This is characterized by increased expression of genes encoding HSPs and chaperone-binding proteins such as *STIP1* and *STI1*. Overexpression of STIP1 has been shown to aggravate α-syn-mediated toxicity in several models, including mouse models [56] and SH-SY5Y cell assays [57]. This supports earlier hypotheses that microglia attempt to clear misfolded protein aggregates in response to proteinopathies, including in PD [51–53, 58]. This process is likely to be energy intensive, as supported by our observations that ATPase regulator activity and other ATP-dependent functions are upregulated in both cell types in PD. These findings suggest that PD microglia and astrocytes are under increased energetic demand, further supported by the upregulation of mitochondrial translation and quality control genes, including *MRPS6*, *MRPL18*, *BNIP3L*, and *HSPE1*. Taken together, our data support the hypothesis that glial cells are under increased energetic stress, possibly leading to a shift in functional priorities. This heightened energy demand may come at the expense of homeostatic or supportive roles, such as neurotransmitter receptor activity, including glutamate receptor signaling in microglia and ion channel-mediated function in astrocytes, which we observed to be downregulated in PD. These patterns are consistent with a model in which chronic mitochondrial and energetic stress drives glial cells to downregulate normal supportive functions in favour of more urgent proteostatic and autophagic processes, particularly in later disease stages.

Interpretation of mitochondrial-related signals from snRNAseq data requires caution, particularly when inferred from broad transcriptomic changes across cell types. Elevated mitochondrial gene expression in sequencing data is often associated with cellular stress or compromised cell integrity during sample preparation and is therefore commonly used as a quality control metric rather than a direct readout of mitochondrial function. Transcriptomic data do not capture key functional parameters such as respiratory capacity, ATP production, membrane potential, or protein import efficiency. In future studies, mitochondrial dysfunction could be more directly assessed using iPSC-derived glial models, through measurements of oxidative phosphorylation and mitochondrial respiration, imaging of mitochondrial morphology or membrane potential, and proteomic profiling of mitochondrial pathways.

Across all three datasets, PD microglia show a broad downregulation of genes associated with resting or surveillant microglia (*P2RY13*, *CX3CR1*, *CH25H*, *CSF2RA,* and *CSF3R*) as well as genes associated with cytokine receptor activity (*IL21R*, *IFI44L*, *IL4R*, and *RSAD2*). Genes associated with lysosomal functions such as *LPL* and *LGALS3* are upregulated, as is *GPNMB*, a known DAM marker. Although *GPNMB* is normally associated with pathways that ameliorate pro-inflammatory signaling [59–61], one study demonstrated a potential role for it in triggering intracellular processes required for pro-activation signalling [62], suggesting a dual role for *GPNMB* in mediating complex microglial states. Importantly, our findings regarding altered inflammation in glia are constrained by the nature of snRNAseq data, as many cytokines and chemokines are predominantly cytosolic or associated with extranuclear compartments and are therefore not fully captured by this technology. Future studies using complementary experimental systems, such as cell culture models, biofluid analyses, and live-cell imaging of lysosomal dynamics under stress, alongside single-cell RNA sequencing, will be important to further resolve these inflammatory and functional changes. Analysis of post-mortem patient data examines tissue at the end stage of disease and, therefore, the end stage of glial dysfunction. Microglia and astrocytes are dynamic cell types with a range of activation states, and an end-stage disease model may only capture the fatigued state of the cells following prolonged activation. Cell culture studies have shown that there is an efficiency curve for microglial clearance of protein aggregates, where it is a protective mechanism at initial stages [58]. However, with increased accumulation and a decline in phagocytotic efficacy, microglia begin releasing fragmented misfolded proteins, leading to a propagation of proteinopathy in a prion-like fashion [13, 50, 63]. A similar role in spreading misfolded protein fragments has also been posited in astrocytes [64]. In astrocytes, altered expression of genes associated with voltage-gated channel functions indicates their role in mediating altered synaptic transmission, likely impacting their communication with other cell types, including microglia and neurons. Reactive states of microglia and astrocytes have been shown to impact each other [65], possibly creating a feedback loop of reactivation that exacerbates neurodegeneration.

Treating these observations as a limited end-stage disease model allows us to reconcile these findings with earlier findings in patients. PET imaging in PD patients has shown increased activation of radioligand-labeled TSPO+ microglia in a sample of PD patients with a range of disease progression measured by the Unified Parkinson’s Disease Rating Scale-III. The study found no correlation between disease stage and microglial activation [12], suggesting that altered states likely precede external diagnostic symptoms of PD. Similar findings in AD [11] and existing review literature [21, 66] suggest that the inflammatory balance mediated by glia is altered *before* the onset of neurological symptoms. Thus, in upcoming studies, it will be important to investigate functional alterations in PD microglia in earlier developmental stages using in vitro models, and in particular the role of these genes in iPSC-derived microglial models of PD.

Finally, public datasets have limited diagnostic and clinical information attached to the sequencing data, thereby limiting the interpretations that can be made regarding clinical outcomes. Due to the nature of the public datasets, we cannot control for factors such as non-demographic patient characteristics, or variance introduced by tissue handling procedures. These effects are inevitable since samples come from a range of biobanks in the USA and the UK and are handled by many people before being processed for sequencing.

## Conclusions

SnRNAseq data from post-mortem PD patients and matched healthy donors allowed us to profile altered microglial and astrocytic functions in the midbrain. Increased expression of chaperone protein binding and protein folding genes suggests a role in clearing protein aggregates by both microglia and astrocytes. Interestingly, decreased expression of genes involved in immune receptor function in microglia implies that late-stage microglia may be fatigued, thereby performing their normal functions with decreased efficacy. Altered expression of genes associated with channel functions in astrocytes suggests that signalling mechanisms may also be disrupted in these supporting cells that often mediate cross-glial communication in the brain. This is supported by the concomitant decreased expression in PD of genes associated with neurotransmitter receptor functions regulating post-synaptic membrane potential. Future studies will explore functional alterations in microglia and astrocytes in earlier disease stages using in vitro models, enabling longitudinal tracking of dynamic changes in inflammation-related cytokine signalling. These analyses will be complemented by biofluid profiling, which, when integrated with sequencing data, will provide a more comprehensive view of glial functional dynamics in PD.

## Electronic supplementary material

Below is the link to the electronic supplementary material.

Supplementary material 1

Supplementary material 2

Supplementary material 3

Supplementary material 4

Supplementary material 5

Supplementary material 6

Supplementary material 7

Supplementary material 8

Supplementary material 9

Supplementary material 10

Supplementary material 11

Supplementary material 12

Supplementary material 13

Supplementary material 14

Supplementary material 15

Supplementary material 16

## Data Availability

The original datasets are available publicly through NCBI Gene Expression Omnibus under GSE178265 [https://www.ncbi.nlm.nih.gov/geo/query/acc.cgi?acc=GSE178265], GSE184950 [https://www.ncbi.nlm.nih.gov/geo/query/acc.cgi?acc=GSE184950], and GSE157783 [https://www.ncbi.nlm.nih.gov/geo/query/acc.cgi?acc=GSE157783]. All scripts used for analysis and data visualization are compiled in a public Github repository, snGlia-PD [https://github.com/indraroy/snGlia-PD].

## Abbreviations

PD: Parkinson’s disease
NDD: Neurodegenerative diseases
DA: Dopaminergic neurons
SNpc: Substantia nigra *pars compacta*
DAM: Damage associated microglia
LBD: Lewy body dementia
SnRNAseq: Single nuclear RNA sequencing
scRNA-seq: Single cell RNA sequencing
GO: Gene Ontology
DGE: Differential gene expression
HSP: Heat shock proteins
