# Supplementary material for: Single nuclear RNA sequencing shows altered microglial and astrocytic functions in post-mortem Parkinson’s disease tissue
Source: BMC Genom Data. 2026 Jul 4;27:81. doi: 10.1186/s12863-026-01462-2 (PMC13625337; doi:10.1186/s12863-026-01462-2)
Supplement: Supplementary file 1 [file 12863_2026_1462_MOESM15_ESM.pdf]

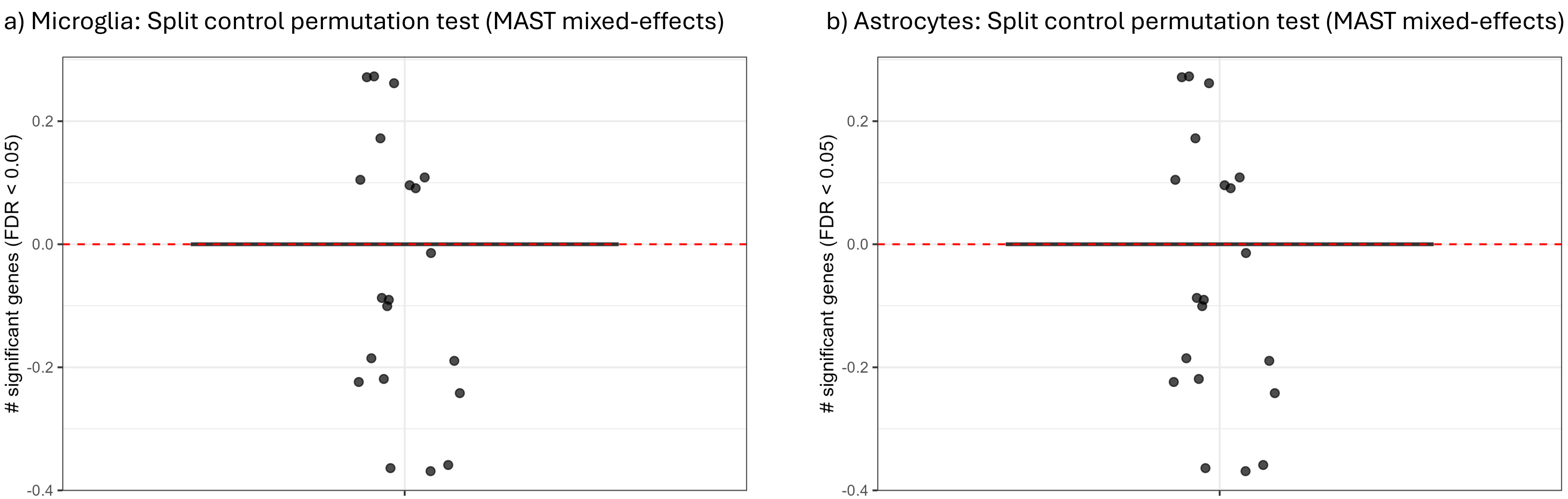
a) Microglia: Split control permutation test (MAST mixed-effects)
b) Astrocytes: Split control permutation test (MAST mixed-effects)
significant genes (FDR < 0.05)
0.2
0.0
-0.2
-0.4
