# Supplementary material for: Single nuclear RNA sequencing shows altered microglial and astrocytic functions in post-mortem Parkinson’s disease tissue
Source: BMC Genom Data. 2026 Jul 4;27:81. doi: 10.1186/s12863-026-01462-2 (PMC13625337; doi:10.1186/s12863-026-01462-2)

kamath mg MG_CECR2_FGL1 gseaMF

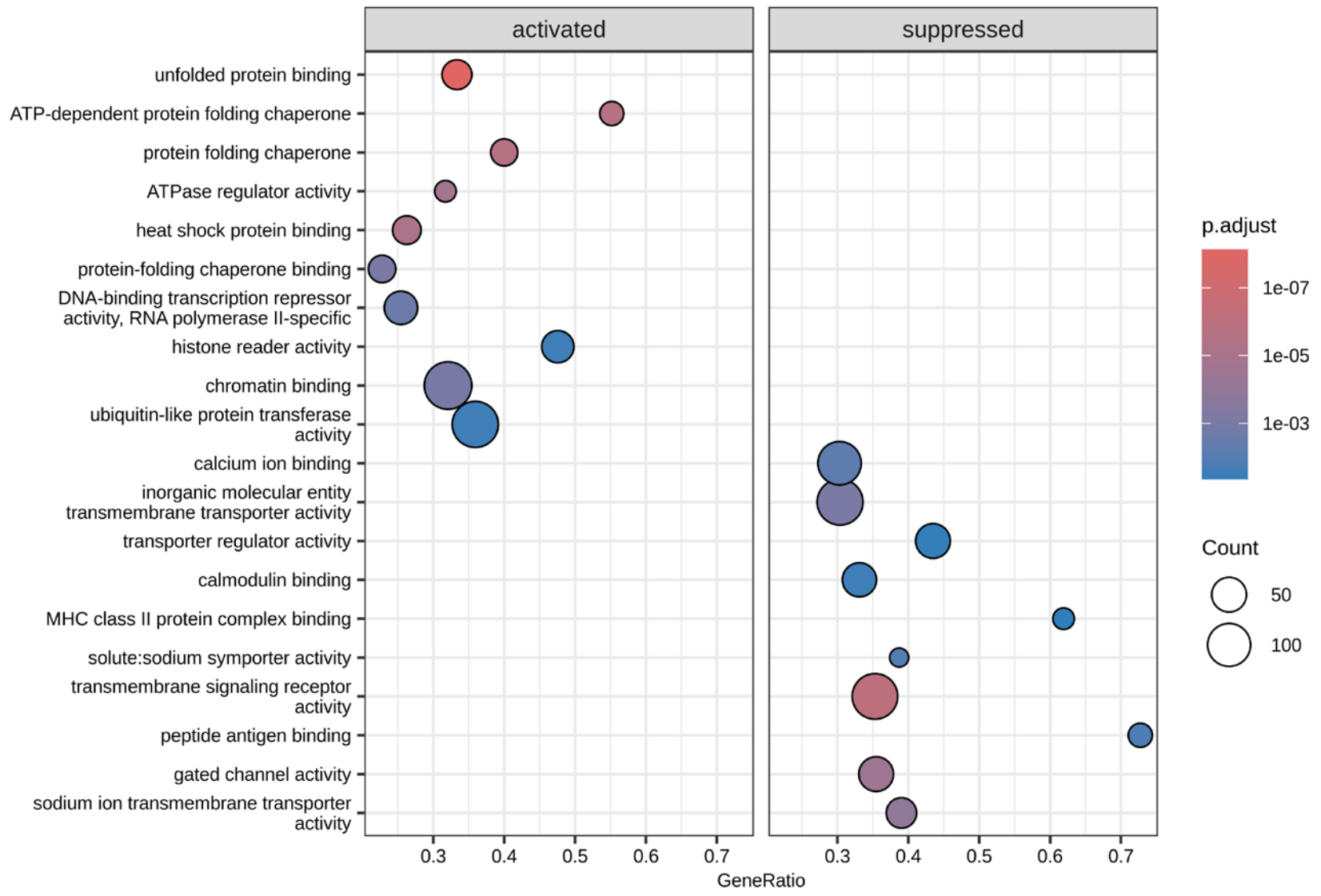

kamath mg MG_GPNMB_LPL gseaMF

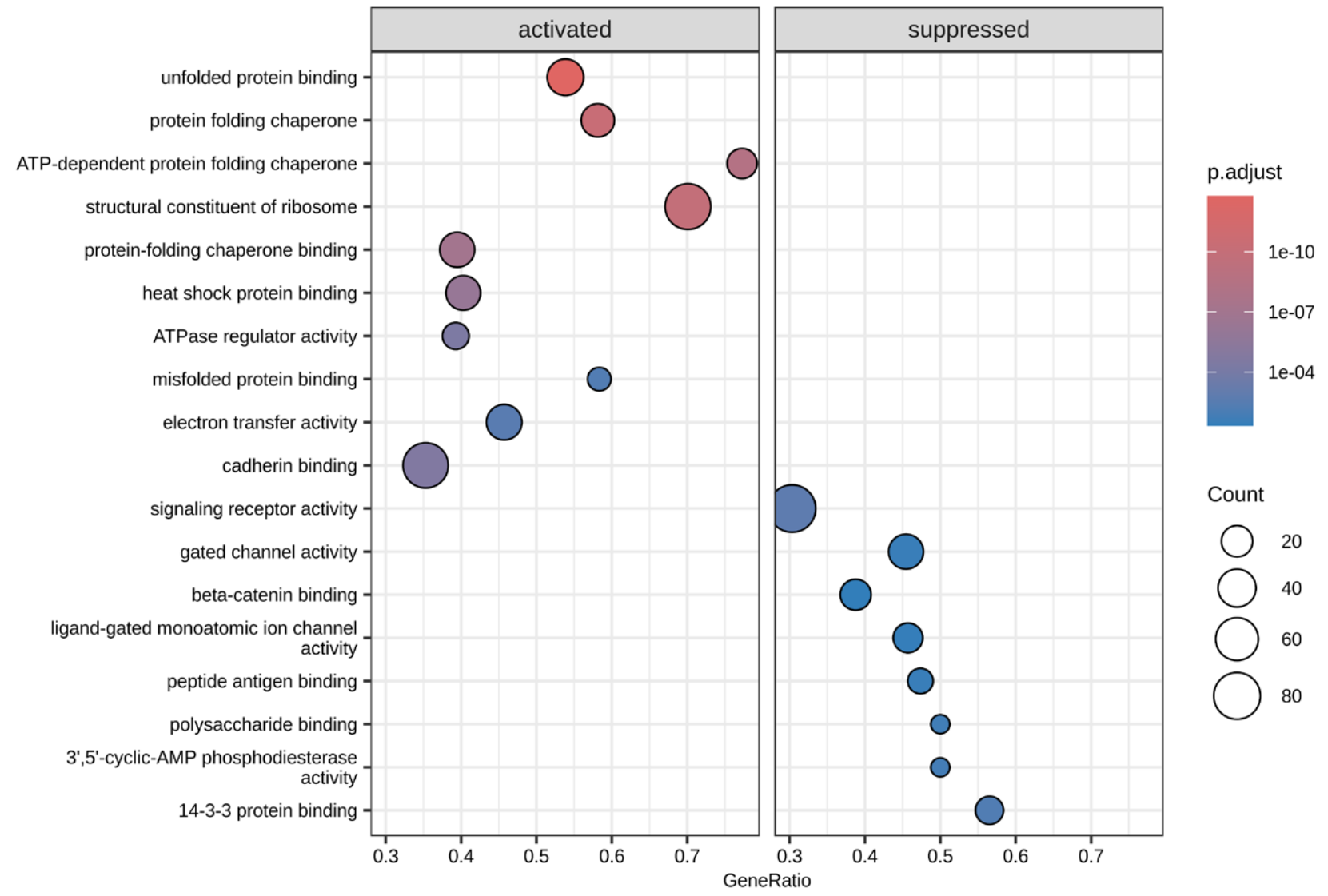

kamath mg MG_GPNMB_SULT1C2 gseaMF

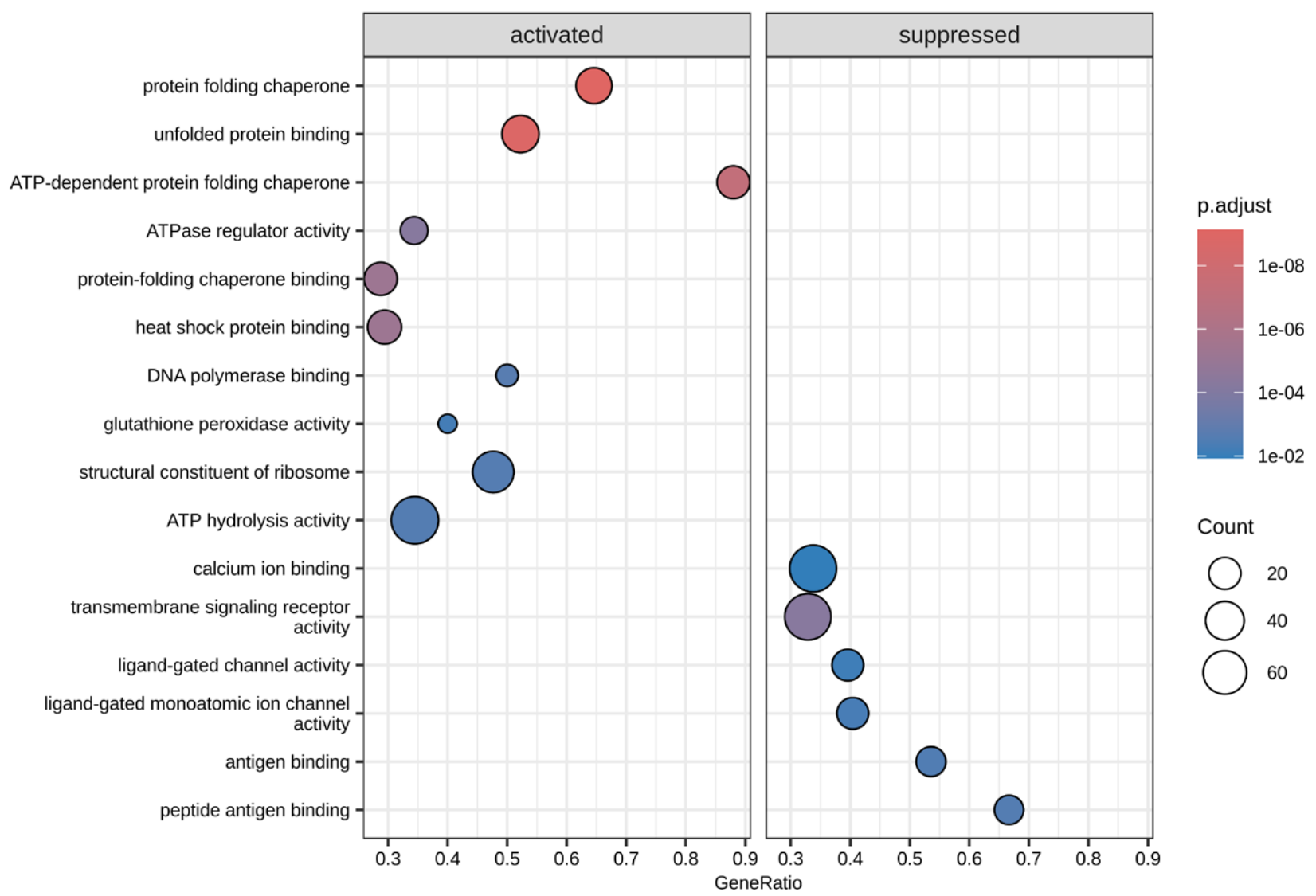

kamath mg MG_OPRM1 gseaMF

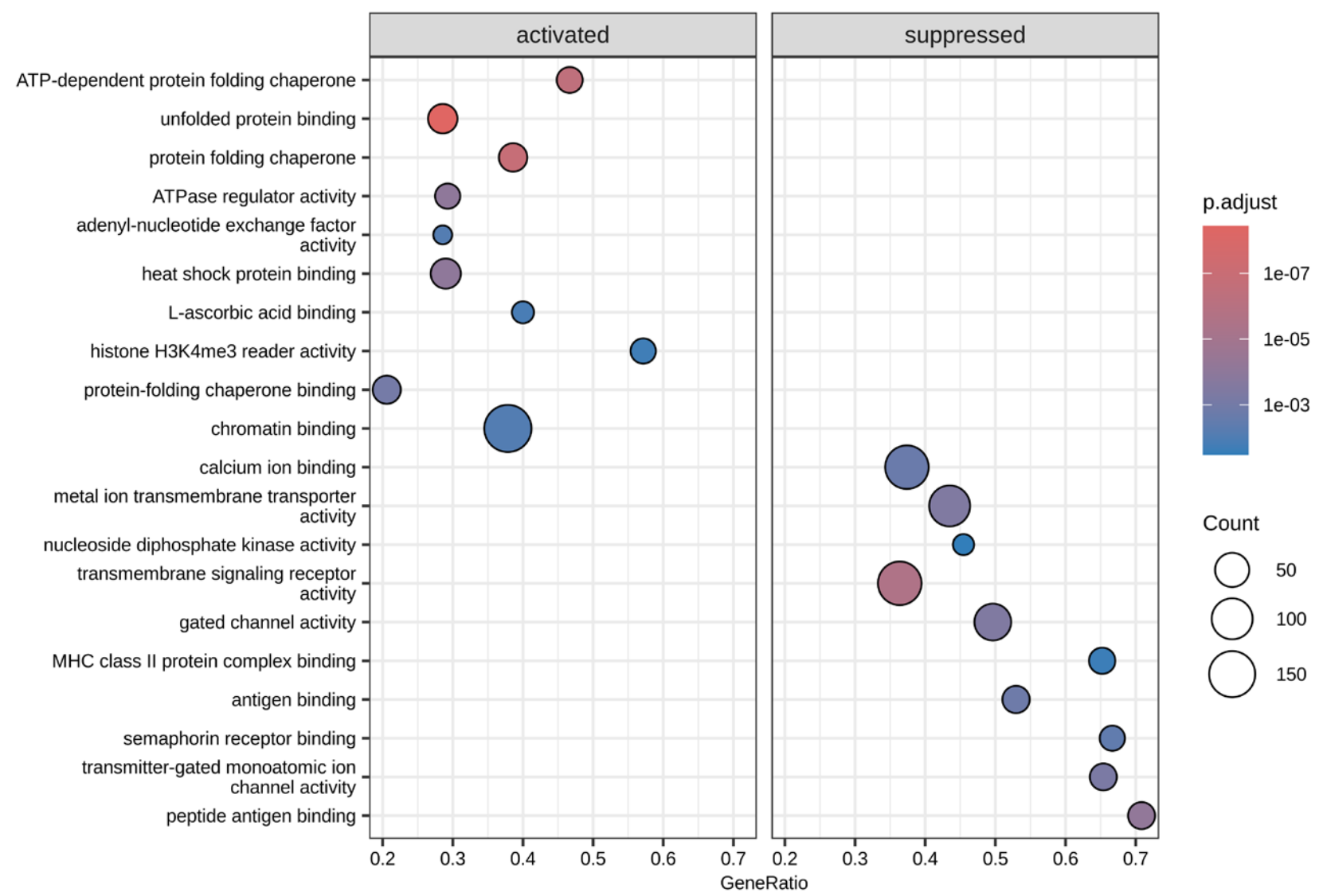

kamath mg MG_TSPO_VIM gseaMF

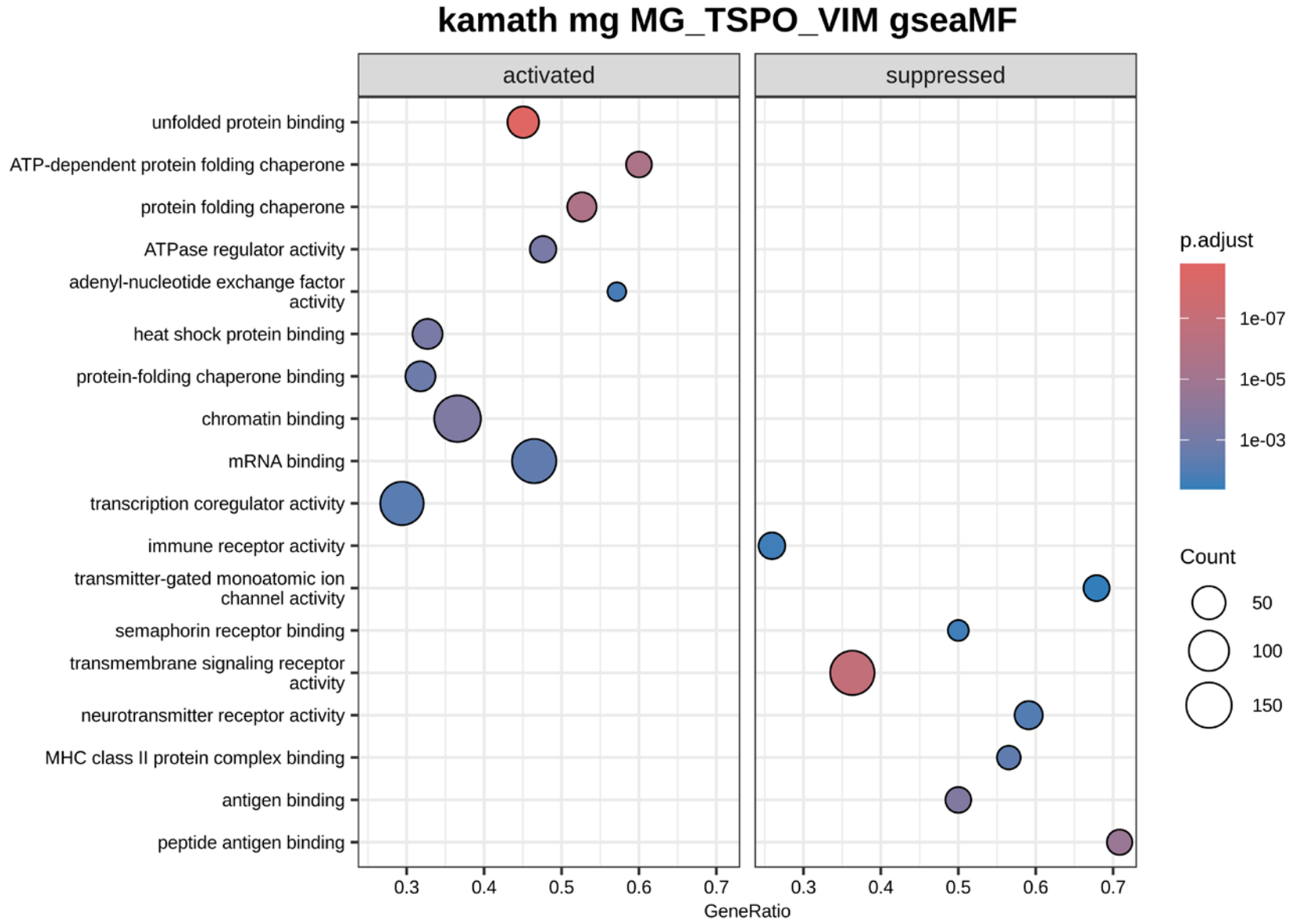

kamath mg Macro_CD200R1 gseaMF

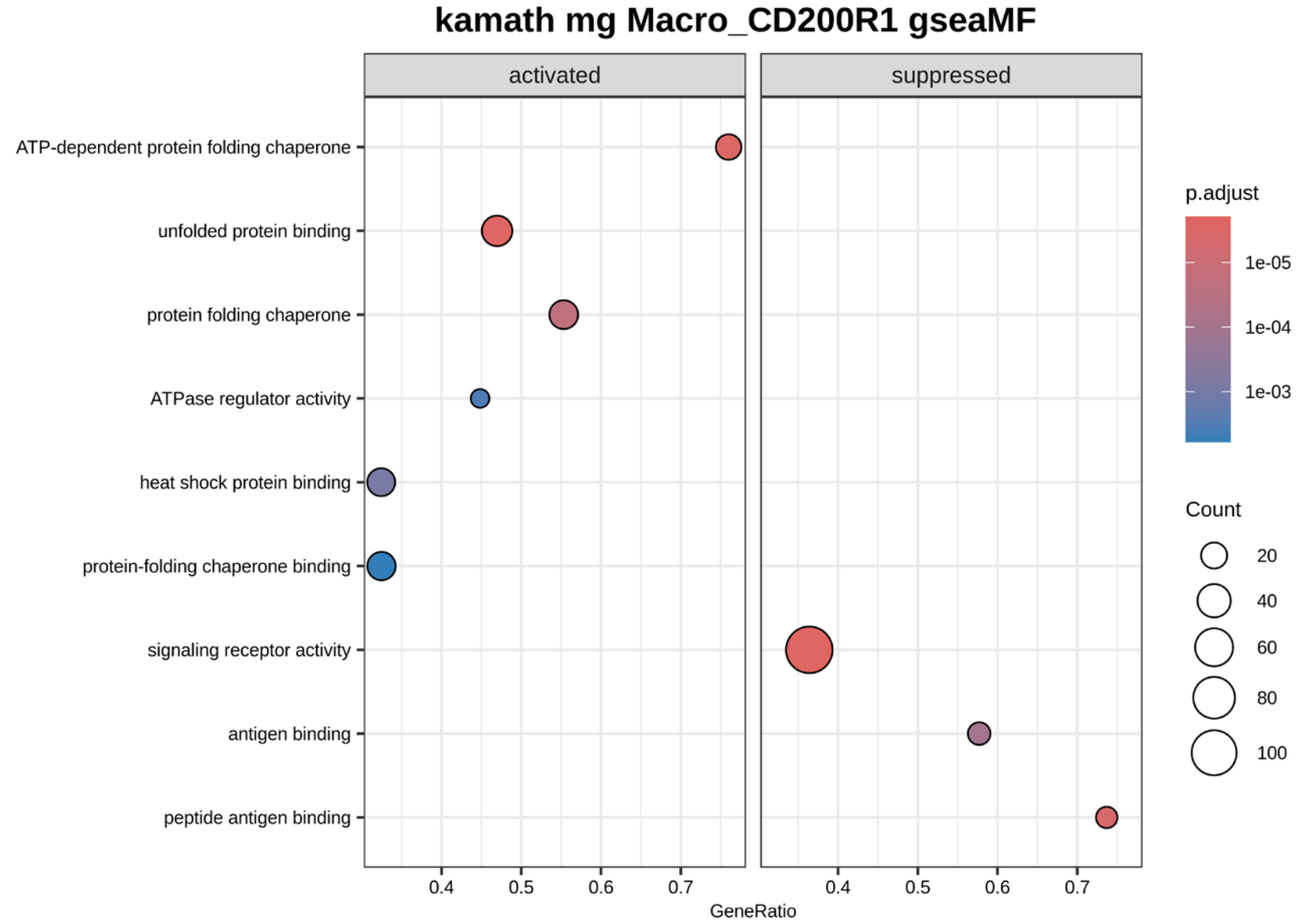

Supplement: Supplementary file 2 [file 12863_2026_1462_MOESM16_ESM.pdf]
